# Strain improvement of *Pichia kudriavzevii* TY13 for raised phytase production and reduced phosphate repression

**DOI:** 10.1111/1751-7915.12427

**Published:** 2016-10-28

**Authors:** Linnea Qvirist, Egor Vorontsov, Jenny Veide Vilg, Thomas Andlid

**Affiliations:** ^1^ Department of Biology and Biological Engineering, Food and Nutritional ScienceChalmers University of TechnologySE‐412 96GothenburgSweden; ^2^ Proteomics Core FacilityGothenburg UniversitySE‐405 30GothenburgSweden

## Abstract

In this work, we present the development and characterization of a strain of *Pichia kudriavzevii* (TY1322), with highly improved phytate‐degrading capacity. The mutant strain TY1322 shows a biomass‐specific phytate degradation of 1.26 mmol g^−1^ h^−1^ after 8 h of cultivation in a high‐phosphate medium, which is about 8 times higher compared with the wild‐type strain. Strain TY1322 was able to grow at low pH (pH 2), at high temperature (46°C) and in the presence of ox bile (2% w/v), indicating this strain's ability to survive passage through the gastrointestinal tract. The purified phytase showed two pH optima, at pH 3.5 and 5.5, and one temperature optimum at 55°C. The lower pH optimum of 3.5 matches the reported pH of the pig stomach, meaning that TY1322 and/or its phytase is highly suitable for use in feed production. Furthermore, *P. kudriavzevii* TY1322 tolerates ethanol up to 6% (v/v) and shows high osmotic stress tolerance. Owing to the phenotypic characteristics and non‐genetically modified organisms nature of TY1322, this strain show great potential for future uses in (i) cereal fermentations for increased mineral bioavailability, and (ii) feed production to increase the phosphate bioavailability for monogastric animals to reduce the need for artificial phosphate fortification.

## Introduction

Phytases are enzymes that degrade phytate by hydrolysing its phosphate groups and simultaneously release its bound or chelated minerals, proteins and/or starches. In order for monogastric animals, including humans, to utilize the nutrients bound to the phytate in food, degradation of phytate is necessary. Monogastric animals do not have phytase enzymes in the intestinal tract, hence phytate degradation needs to be mediated by external enzymes. Phytate degradation can be achieved in different ways, for instance during food fermentation by phytase‐active microorganisms (De Angelis *et al*., 2003; Rizzello *et al*., 2010), through addition of commercial phytase solutions (mainly in the feed industry) (Dersjant‐Li *et al*., 2014) or by endogenous phytases present in the food or feed raw materials (Leenhardt *et al*., 2005). In the feed industry, phytase solutions are added to the feed with the main goal of releasing phosphate, thereby reducing the need for artificial phosphate fortification and reducing the subsequent eutrophication issue. For pig feed applications, a phytase having a pH optimum around 3.5 is desirable, as this is the approximate pH in the stomach of pigs (Kim *et al*., 2006), and can allow continuous phytate degradation inside the stomach also after ingestion of the feed. In human nutrition, the focus is on increasing the mineral and protein bioavailability from the food. Addition of commercial phytase solutions is currently not applied in human food production, mainly due to the fact that all commercial phytase producing organisms today are genetically modified organisms (GMO), which is commonly not accepted for human food production. Phytate degradation in food is instead mediated mainly by fermentations using phytate‐degrading microorganisms, or during the food processing by the endogenous phytases in the food matrix.

One example of a food fermentation of recent increasing scientific and public interest is sourdough. In sourdough fermentations, both yeasts and lactic acid bacteria are present, and contribute to both the organoleptic and nutritional properties of the product, for example through phytate degradation and mineral release (Nielsen *et al*., 2007; Pable *et al*., 2014; Caputo *et al*., 2015). Previous studies have reported isolation of several different yeast species from sourdough (Meroth *et al*., 2003; Pulvirenti *et al*., 2004; Nuobariene *et al*., 2012) where *Pichia kudriavzevii* is one of the often isolated species. In our previous work (Hellström *et al*., 2012; Hellstrom *et al*., 2015b,b), one strain of *P. kudriavzevii*, TY13, originally isolated from the traditionally fermented Tanzanian food called Togwa (Hellström *et al*., 2010), was investigated for its high phytate‐degrading capacity. Although several yeasts are known to produce phytase enzymes (Kaur *et al*., 2007; Hellström *et al*., 2010), the enzymes are in most cases exported only into the periplasmatic space, leaving the enzymes trapped inside the yeast cell wall. Our previous work on *P. kudriavzevii* TY13 revealed, however, this strain's impressive ability to release non‐cell‐bound phytases also to the surrounding medium, from young growing populations (i.e. not by leaking from lysing old cells) under certain cultivation conditions (Hellstrom *et al*., 2015b,b). Released enzymes in the surrounding media may (i) significantly increase the interactions between the substrate (phytate) in the food matrix and the phytase enzyme, thereby increasing the overall phytate degradation and mineral release and (ii) greatly ease the product recovery during industrial production of phytase solutions. Furthermore, the TY13 strain was able to rapidly degrade phytate also at moderately high concentrations of phosphate (Pi) in the surrounding medium, which has previously shown to inhibit the phytase expression by yeast (Andlid *et al*., 2004). In addition, recent data from our laboratory (unpublished) also revealed efficient degradation of phytate during fermentation of a model bread dough using TY13, indicating the strains promising capacity to be used in future starter cultures and/or commercial bread production.

As the phytase activity from the promising strain TY13 was still to some extent regulated in response to the medium composition in our previous study (Hellstrom *et al*., 2015b,b), the present study was undertaken to further evolve TY13 to create strain(s) with (i) improved phytate degradation at high surrounding Pi levels, (ii) increased phytate degradation per biomass and, preferably (iii) increased ratio of exported non‐cell‐bound phytases. As GMO are commonly not accepted for use in food production, and not well received by consumers, this study employs the alternative method of random mutagenesis induced by UV irradiation, followed by selection of positive mutant strains. As the nature of the mutation(s), i.e. the location and type of mutation, is not known using this random mutagenesis method, an important aspect during the evaluation of mutant strains is to ensure sustained phenotypic traits. For successful implementation of the mutant strain in industrial or household settings, maintained growth capacity under different conditions is of high importance.

In this study, we present the significantly improved yeast strain, TY1322, originating from TY13. This study presents strain mutagenesis and selection, phenotypic characterization the strains, characterization of the biomass‐bound phytase and finally the purification and characterization of the released non‐cell‐bound phytase.

## Results

### Mutagenesis and isolation of improved strains of *P. kudriavzevii* TY13

Exposure of UV at 254 nm for 18 s was chosen to achieve about 60% survival rate. The mutagenesis was performed in two consecutive rounds, using strain *P. kudriavzevii* TY13wt (wild type) as parental strain in the first round, and strain TY1310 in the second round.

From the *first* round, a total of 6653 colonies were examined for blue colour formation on 5‐Bromo‐4‐chloro‐3‐indolyl phosphate (BCIP) agar plates. The screening resulted in selection of 33 colonies with presumably increased phosphatase activity, based on stronger blue colour formation on the BCIP plates. Screenings of the 33 selected putative mutants in liquid cultures of PM_NoPi_ and PM_HPi_ led to the final selection of a strain annotated TY1310 as the most prominent one (data not shown).

In the *second* round of mutagenesis, using strain TY1310 (from the first round) as parental strain, a total of 8109 colonies were investigated on the BCIP agar plates. From those, 89 colonies were initially selected for a second investigation on BCIP plates, resulting in a final selection of 21 colonies (having stronger blue colour formation) for further screenings.

### Screenings for strains with improved phytate‐degrading capacity

**Figure 1:**
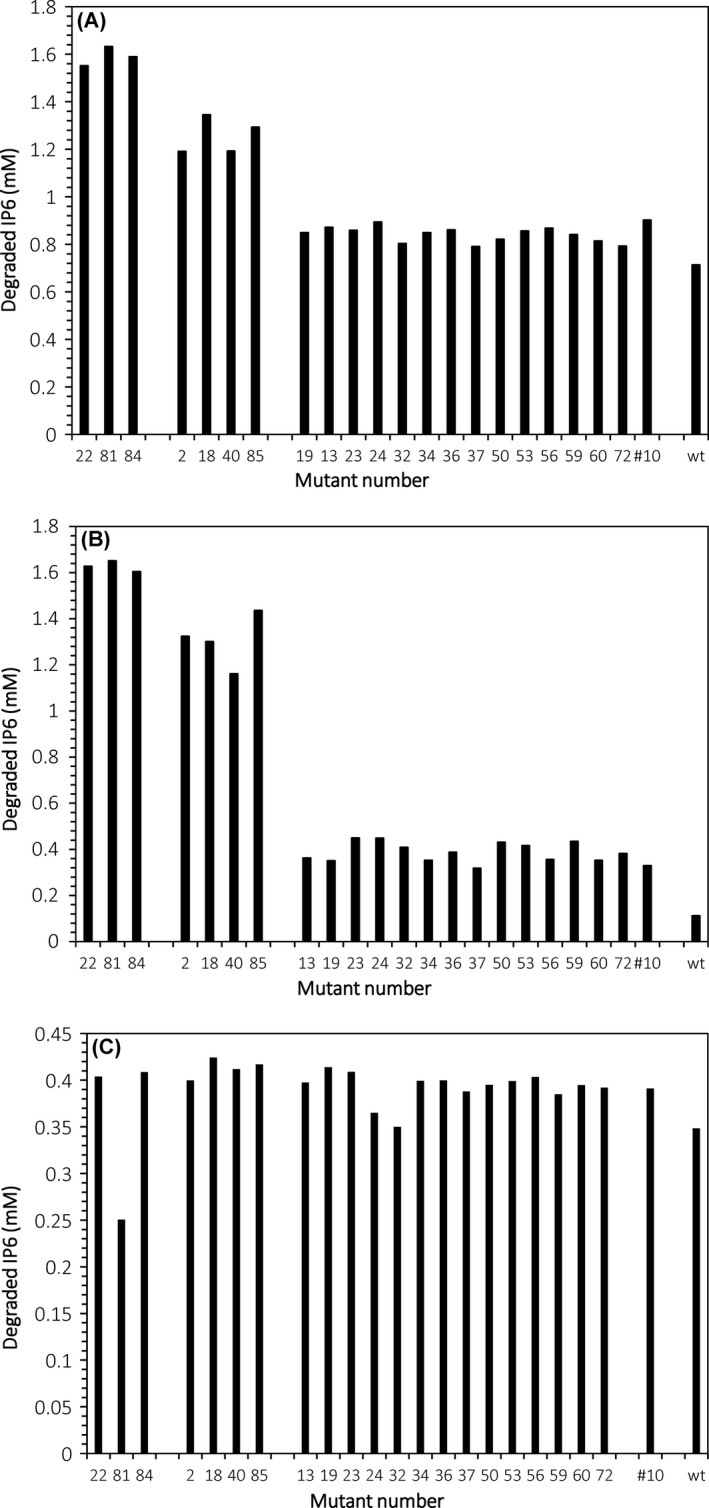
Screenings of the 21 putative mutants of the second‐round mutagenesis, the wild‐type strain TY13 (wt) and the second‐round mutagenesis parental strain TY1310 (number 10). (A) IP_6_ degradation in single liquid cultures in PM_N__oPi_ medium (YNB w/o phosphate, IP_6_ 3 g l^−1^, glucose 20 g l^−1^ in succinate buffer). The presented data represent degradation after 6 h of incubation. (B) IP_6_ degradation in single liquid cultures in PM_HP__i_ medium (YNB w/o phosphate, IP_6_ 3 g l^−1^, glucose 20 g l^−1^, Pi 3.5 g l^−1^ in succinate buffer). The presented data are after 6 h of incubation. (C) Screening for released non‐cell‐bound phytase activity in a secretion‐inducing medium (YNB w/o phosphate, yeast extract 10 g l^−1^, glucose 20 g l^−1^ in succinate buffer pH 5.5). The assay is performed on cell‐free supernatants from 9 h old cultures, and the presented data are from 15 min of assay time.

First, single liquid cultures of the 21 putative mutants from the second round of mutagenesis, together with the two parental strains TY13wt and TY1310, were assessed for phytate degradation in PM_NoPi_ (phosphate‐free phytate medium) and PM_HPi_ (high‐phosphate phytate medium). The putative mutants fell into three groups based on amount degraded IP_6_, showing high (number 22, 81, 84), medium (number 2, 18, 40, 85) or low (number 13, 19, 23, 24, 32, 34, 36, 37, 50, 53, 56, 59, 60, 72) phytate degradation (Fig. 1A and B).

A large group of the putative mutants (number 13, 19, 23, 24, 32, 34, 36, 37, 50, 53, 56, 59, 60, 72) together with the two parental strains, TY1310 and TY13wt, showed inhibited phytate‐degrading capacity at high Pi levels (Fig. 1B). In the Pi‐free medium (Fig. 1A), this group still showed clearly lower phytate‐degrading capacity compared with the other two groups. The original strain, TY13wt, consistently showed the lowest phytate‐degrading capacity at the prevailing conditions, and the second parental strain, TY1310, was found among the low‐phytate‐degrading group of putative mutants.

Second, the 21 putative mutants were further assessed for secretion of non‐cell‐bound phytase in secretion‐inducing medium (SIM) and in a non‐secretion‐inducing medium (NSIM). In SIM, none of the mutants demonstrated any obvious improvement in non‐cell‐bound phytase activity compared with the parental strains TY13wt and TY1310 (Fig. 1C). Furthermore, none of the putative mutants showed secretion of non‐cell‐bound phytase in NSIM (data not presented).

From the screenings, strains TY1322, TY1384 and TY1381 showed the most prominent results in terms of phytate‐degrading capacity and reduced phosphate repression. From the growth assessment, strain TY1381 showed impaired growth at several conditions (data not shown). Strain TY1322 was finally selected for further characterization.

### Characterization of phytase‐active mutant strains TY1310 and TY1322

The mutant stability of strains TY1310 and TY1322 was assessed by cultivation of the strains for several generations on non‐selective medium (yeast extract peptone dextrose, YPD), followed by assessing the phytase activity. At no occasion were there any fluctuations in phytase activity observed from the strains, indicating that the mutation obtained is stable in the strains.

**Figure 2:**
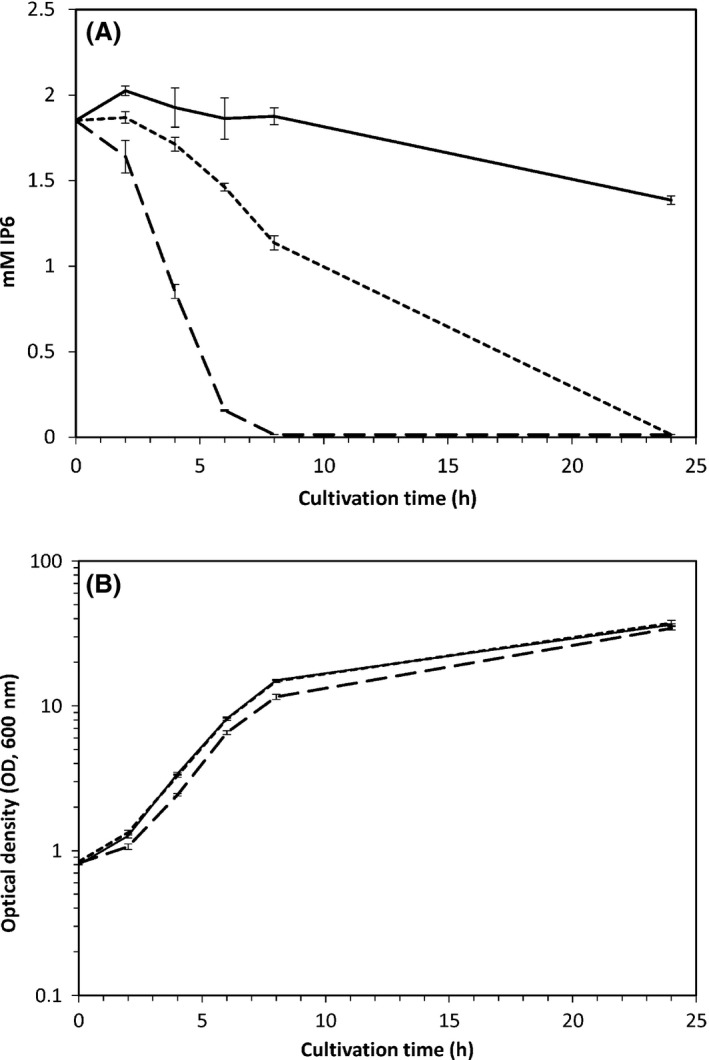
Phytate degradation and growth (optical density at 600 nm) for strains TY13wt (solid line), TY1310 (dotted line) and TY1322 (dashed line), cultivated in PM_HP__i._ All data are means of triplicate cultures with standard deviation presented. Panel A shows the phytate degradation (mM) and panel B shows the growth as measured by optical density (600 nm) during 24‐h cultivation.

To more thoroughly compare the two strains TY1310 and TY1322 with the wild‐type strain TY13wt, the growth and phytate‐degrading capacities of the strains were assessed by cultivations in PM_HPi_ medium. Phytate‐degrading capacity and growth performance were assessed. As seen (Fig. 2A and B) strain TY1322 showed almost complete phytate depletion at 6 h of cultivation (1.69 mM phytate degraded), while strain TY1310 showed only 0.39 mM phytate degradation and TY13wt showed no degradation. The growth capacity is largely maintained in the two improved mutant strains compared with the wild‐type strain.

Furthermore, the degradation was also evaluated in PM_NoPi_ medium, where strain TY1322 showed complete phytate degradation (1.87 mM) at 6 h of cultivation and 1.12 mM degradation at 4 h of cultivation. At 6 h and 4 h in the same medium, strains TY1310 showed 0.82 mM and 0.17 mM phytate degradation, and strain TY13wt showed 0.68 mM and 0.07 mM phytate degradation respectively.

**Figure 3:**
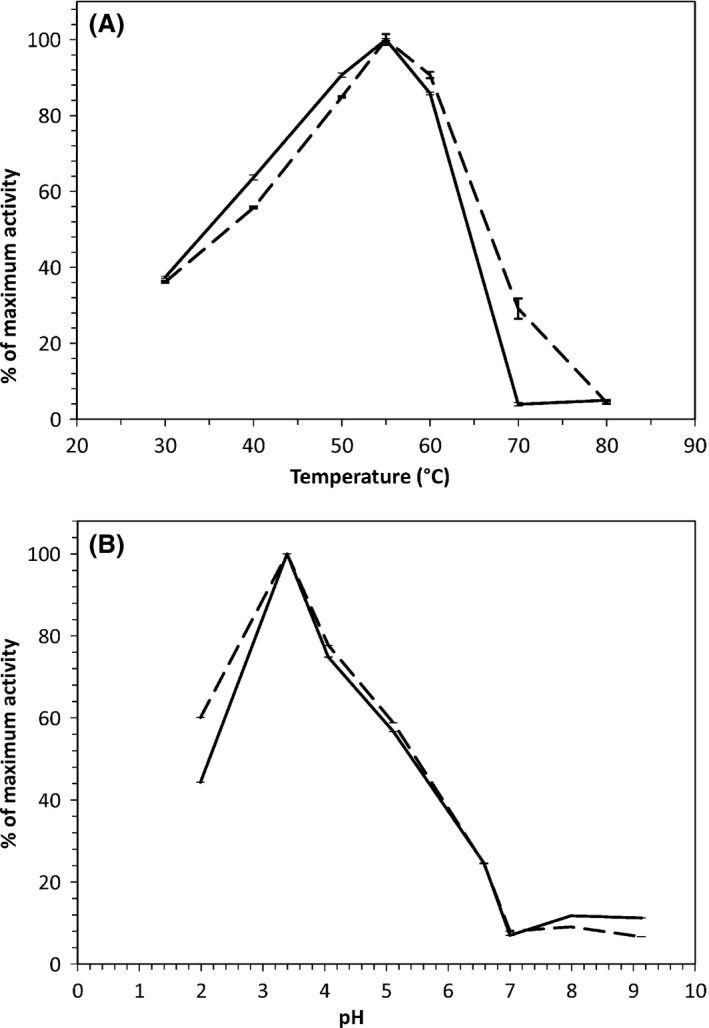
Phytase activity from washed yeast biomass at different temperatures from 30°C to 80°C (on the left), and at different pH from 2 to 9 (on the right), strain TY13wt is presented by a solid line and TY1322 by a dashed line.

The temperature and pH optima for the strains phytase activities were determined, revealing a phytase activity optimum at 55°C for both TY1322 and TY13wt (Fig. 3A). The phytase activity at different pH for the biomass‐associated phytase of TY13wt and TY1322 showed a pH optimum at 3.5 (Fig. 3B).

**Figure 4:**
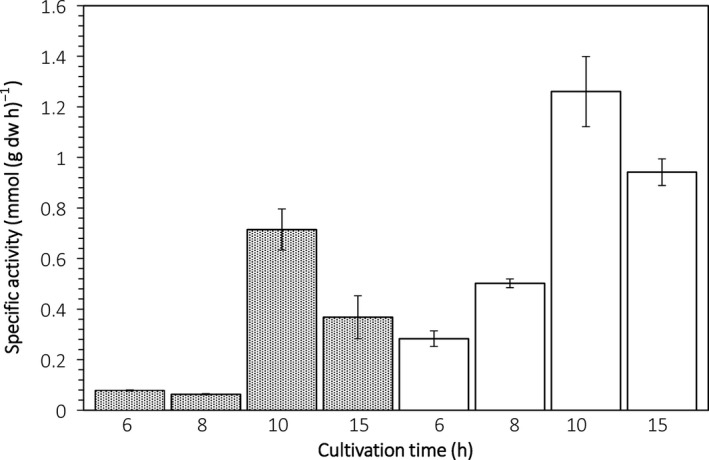
Biomass‐specific phytase activity during cultivation of strains TY13wt (grey bars) and TY1322 (white bars) in a high‐phosphate (3 g l^−1^) and high‐phytate (3 g l^−1^) medium. Samples were taken at 6, 8, 10 and 15 h and the biomass specific activity is presented as mmol degraded IP_6_ per g dry weight biomass and hour of enzymatic reaction.

To investigate the cell‐bound phytate‐degrading capacity from the viable yeast cells of strains TY13wt and TY1322, biomass was harvested after 6, 8, 10 and 15 h of incubation in a Pi‐rich and IP_6_‐rich medium. The biomass‐bound activity by strain TY1322 was consistently higher than that of the parental strain TY13wt (Fig. 4). The wild‐type strain showed no phytate degradation during the first 8 h of cultivation, whereas strain TY1322 showed immediate phytate degradation already at the first time point (6 h). At 8 h of cultivation, the biomass‐bound activity by TY1322 is 1.26 mmol g^−1^ h^−1^, which is about 8 times higher compared with TY13wt. Furthermore, both strains show a peak in biomass‐bound activity at 8 h of cultivation under those conditions.

### Purification and characterization of TY13 phytase

The secreted non‐cell‐bound phytase was concentrated and purified by fractionation on Sephadex G75 gel column. Samples from each step of the purification process were analysed for phytase activity (U ml^−1^) and protein content (mg ml^−1^) to determine the yield and degree of purification. Table 1 presents the protein content and the phytase activity of the samples from each of the purification steps.

**Table 1:** Summary of purification of phytase from *Pichia kudriavzevii* TY13wt and TY1322

Sample	Total protein (mg)	Total activity (mU)	Specific activity (U g^−1^)	Purification (fold)	Yield (%)
TY13wt					
Culture filtrate	2810.06	55 610	19.79	1.00	100
Amicon concentrate	85.84	15 210	177.21	8.96	27.36
Spin filter concentrate	43.95	12 200	277.66	14.03	21.95
Sephadex G75	6.15	12 950	2105.69	106.41	23.29
TY1322					
Culture filtrate	3260.05	62 750	19.25	1.00	100
Amicon concentrate	101.85	16 140	158.43	8.23	25.72
Spin filter concentrate	80.04	12 330	154.03	8.00	19.65
Sephadex G75	5.54	12 540	2266.42	117.75	19.99

The protein content and the phytase activity of the analysed fractions for TY13wt and TY1322 were investigated (Fig. S1). The analysis of protein content yielded three main peaks, in fraction 4–5, fraction 7 and fraction 10–13, with a shoulder peak in fraction 15 (Fig. S1). For both strains, the phytase activity was found in the first protein peak, corresponding to fractions 3, 4 and 5, which were pooled followed by determination of phytase activity and protein content of the pooled sample. Fraction number 6 was not pooled, even though it shows phytase activity, as this fraction possibly also contained the proteins corresponding to the second peak in the chromatogram.

The pooled respectively samples of TY13wt and TY1322 were denaturated with mercaptoethanol and run on a polyacrylamide gel along with a known size ladder. The phytase size was estimated to be 120 kDa for both samples, there were no larger protein bands appearing on this gel.

High‐resolution, high‐mass accuracy proteomic analysis of the respective pooled sample from TY13wt and TY1322 confirmed the identity of phytase proteins in both samples. The genomic database for *P. kudriavzevii* from the Uniprot repository contains four sequences with the RHGXRXP sequence motif, which is characteristic for phytases. The three of four phytase sequences were confidently identified in both purified samples with 1–14 unique peptides at 1% false discovery rate, suggesting that the three sequences are expressed in both samples (Table S1). Proteomic analysis suggests that the phytases are plausibly the main components of both samples. The protein sequence coverage is very similar between the TY13wt and the TY1322 sample, ranging from 19% to 37% for different phytases. However, such an experiment cannot prove or contest the potential sequence differences for each of the phytases between the strains, as the strain‐wise genomic sequencing information is not available.

The purified phytase samples of TY13wt and TY1322 respectively were assessed for temperature and pH optima. The temperature optimum was 55°C (Fig. S2A) for the purified phytase of both TY13wt and TY1322, and there were two pH optima (Fig. S2B) at pH 3.5 and 5.5. The purified phytase showed much broader tolerance towards both pH and temperature, as compared to the assay of the cell‐bound enzymes (Fig. 3A and B).

**Figure 5:**
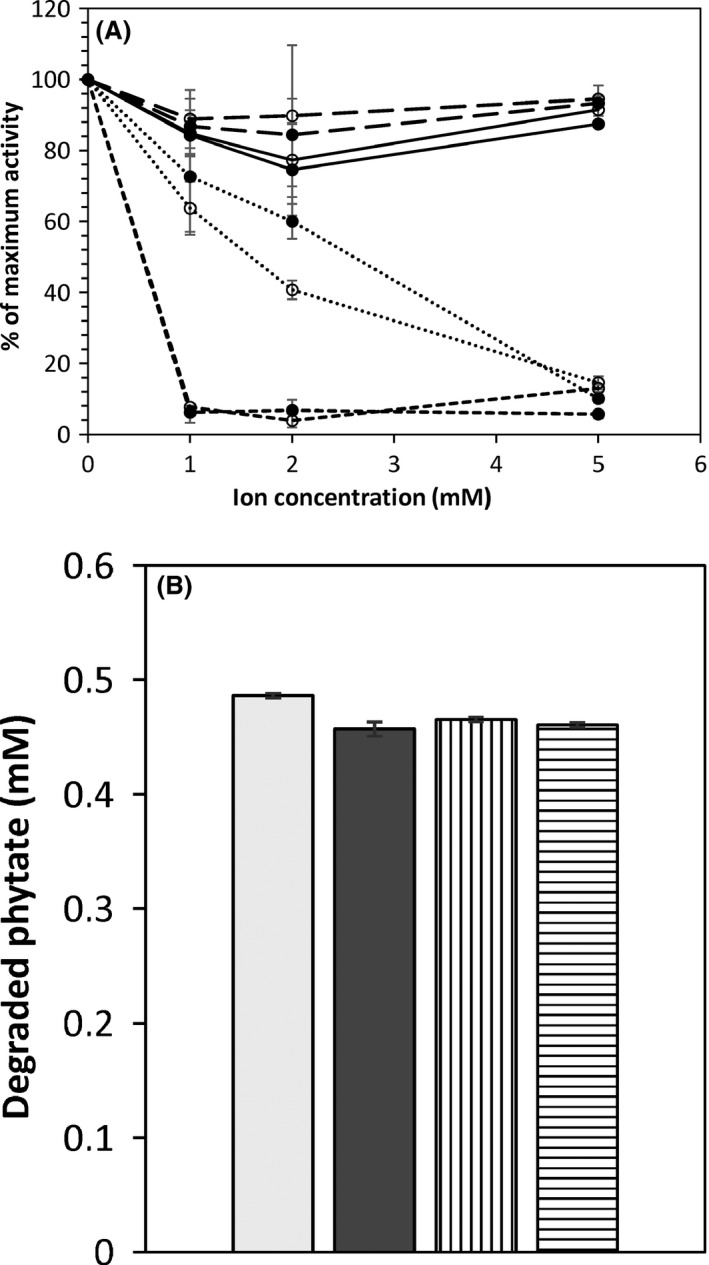
Relative phytate‐degrading capacity (% of maximum activity) of the purified phytase from strain TY13wt (○) and TY1322 (●) during the presence of iron (short dashed line), copper (dotted line), calcium (long dashed line) and magnesium (solid line), presented in (A). (B) It shows the amount of degraded phytate from the purified phytase of strain TY13wt and TY1322 during phytase assay at the presence of phosphate (3.5 g l^−1^) or without the presence of phosphate; TY13wt in high‐phosphate assay (white bar), TY13wt in phosphate‐free assay (grey bar), TY1322 in high‐phosphate assay (vertically striped bar) and TY1322 in phosphate‐free assay (horizontally striped bar).

The phytase samples were further assessed for its phytase activity in the presence of various metal ions and at high levels of phosphate. Both TY13wt and TY1322 phytases showed the same response in activity towards the tested ions; more or less no influence from Ca^2+^ and Mg^2+^ at any of the tested concentrations, an almost linearly increased inhibition from Cu^2+^ with increasing ion concentration and a complete inhibition from Fe^2+^ already at 1 mM concentration (Fig. 5A).

The purified phytase showed no inhibition in phytate‐degrading capacity at high levels of phosphate (3.5 g l^−1^) in the assay mixture compared with the activity found in the phosphate‐free assay mixture (Fig. 5B).

To assess the phytase temperature stability, the purified phytase from strain TY1322 was incubated at the temperatures 55°C, 65°C, 75°C, 85°C and 95°C for 10 respectively 60 s. The results revealed no decrease in phytase activity after 10 s at 65–85°C (above 99% maintained activity compared with the positive control). Incubation at 95°C resulted in 83% maintained activity at 10 s of incubation. After 60 s of incubation, only the sample treated at 65°C maintained any activity, corresponding to 47% of the initial activity.

### Phenotypic characterization of yeast strains TY13wt and TY1322

**Figure 6:**
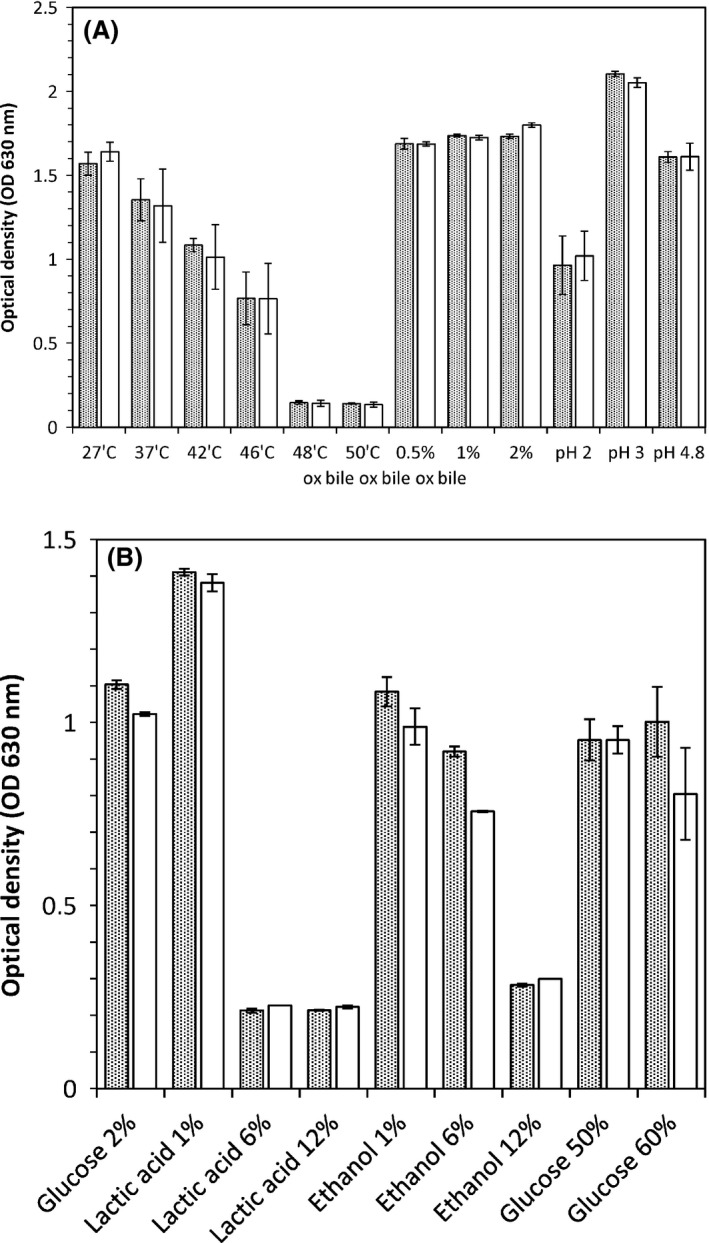
(A) It shows the growth of strain TY13wt (dark bars) and TY1322 (white bars) at different temperatures from 27°C to 50°C (in YPD of pH 6.5), in the presence of ox bile from 0.5% to 2% (in YPD with pH 6.5, at 37°C) and at pH from 2 to 4.8 (at 37°C). Growth was assessed after 3 days on incubation as optical density at 630 nm. (B) It shows the growth of strain TY13wt (dark bars) and TY1322 (white bars) in YP based media with different levels of lactic acid, ethanol or glucose. All incubations were done at 30°C and growth was assessed after 2 days on incubation as optical density at 630 nm.

Growth (determined as optical density) was assessed under acidic conditions, in the presence of ox bile and at elevated temperatures. Those experiments were carried out in duplicate cultures for 3 days of incubation; the results are presented in Fig. 6A.

The two strains, TY13wt and TY1322, showed similar temperature tolerance, being able to grow up to 46°C, albeit the growth was successively impaired with the temperature raise (Fig. 6A). Both strains grew well in the presence of ox bile up to 2% (w/v) and both were able to grow in acidic conditions down to pH 2 (Fig. 6A). The growth was however inhibited at pH 2 and the cultures reached only about 50% of the cell density found at pH 3. Our data suggest that those two strains of *P. kudriavzevii* prefer lower pH for growth, reaching the highest cell density in a medium of pH 3.

Furthermore, growth assessment was also done to investigate utilization of different carbon sources, growth in ethanol or lactic acid and to test the osmotic stress tolerance by cultivation in high‐glucose media. All experiments were again carried out in duplicate cultures, but this time for 2 days of incubation, the results are presented in Fig. 6B.

The two strains TY13wt and TY1322 were able to grow in a medium containing yeast extract (1%), peptone (2%) with either ethanol up to 6%, or with lactic acid at 1% (Fig. 6B). The strains were also tolerant to osmotic stress as induced by glucose concentrations up to 60%.

The strains did not show growth on other carbon sources than glucose in this setup (data not shown). Neither of the strains showed strong resistance towards oxidative stress, having a growth inhibition zone (i.e. the radius from the H_2_O_2_ disc to the growing cells, indicating the zone of inhibition) with a diameter of 17 mm for TY13wt and 15 mm for TY1322.

## Discussion

This study presents the successful mutagenesis of the phytate‐degrading yeast *P. kudriavzevii* TY13 resulting in the improved strain *P. kudriavzevii* TY1322 with strongly increased phytate‐degrading capacity. In addition to superior phytase production, the strain also has other phenotypic properties suitable for applications in food and feed industries.

The improved strain was achieved by random UV mutagenesis and subsequent selection of improved phosphatase positive mutants based on colour development on an agar‐based medium. The selection of phosphatase positive strains successfully also led to the isolation of phytase‐active strains. Our results clearly show improved phytate‐degrading capacity of the improved strain TY1322 over the wild‐type strain TY13wt and the intermediate parental strain TY1310.

There was a very small growth impairment in strain TY1322 at certain conditions (Fig. 2B) which is not an unexpected trade‐off considering that this strain must use a larger part of its available energy for phytase biosynthesis, and hence less for biomass formation, something that has been shown also in previous work (Veide and Andlid, 2006). However, this growth impairment was more or less a negligible side effect in comparison with the strong positive outcome being the essentially improved phytate‐degrading capacity (Fig. 2A).

The phytase activity of strain TY1322 was not repressed at the high phosphate concentrations used in this work (3.5 g l^−1^), as opposed to the wild‐type strain at the same conditions. The strain TY1322 shows promising results also in comparison to other studies on phytases and phosphate repression, for example the phytase from *Sporotrichum thermophile* (Singh and Satyanarayana, 2008) which showed a significant repression on phytase production already at 0.25% phosphate concentration. The superior nature of strain TY1322 was further underlined by a much higher phytase activity from its biomass, compared with TY13wt in a high‐phosphate medium (Fig. 4). The phytase activity from TY13wt biomass was more or less undetectable until 10 h of cultivation, which is probably an effect of the surrounding phosphate levels still being too high for the yeast to actively express phytases. As the phosphate levels in the surrounding medium then decreased during prolonged incubation time, the phytase activity could be detected at the later stages of incubation. For the biomass of strain TY1322 on the other hand, phytase activity was detected already at 6 h of incubation, and after 8 h of cultivation, the activity by biomass of strain TY1322 was about 8 times higher compared with the wild‐type biomass.

Other studies on improvement of microbial phytase activity has used optimization of cultivation conditions as the method for improvement and achieved, for example 3.75‐fold improvement in *S. thermophile* (Singh and Satyanarayana, 2008) and 10‐fold improvement in *Saccharomyces cerevisiae* (Ries and Alves Macedo, 2011). This grade of improvement highlights the outcome of our strain improvement by mutagenesis, being an eightfold improvement, and further opens up for future potential improvement by cultivation optimization.

The phytase from strains TY13wt and TY1322 was concentrated and purified by filtration methods and Sephadex chromatography. This methodology allows a very high degree of purification, but cannot guarantee complete purification as other same sized proteins may be present in the final pooled sample. In this work, we refer to the highly purified and pooled sample as ‘purified’. The purified non‐cell‐bound enzyme showed two pH optima, at 3.5 and 5.5, while the pH optimum for the cell‐associated enzyme (incubation done with washed biomass) was 3.5. The reason for the different pH optima still remains unknown to us, but it may be hypothesized that the presence of the biomass in various ways (chemically or physically) may affect the activity of the phytase. However, the pH optimum at 3.5 correlates well with the pH of the pig intestine (Kim *et al*., 2006), making the phytase produced by *P. kudriavzevii* TY1322 very suitable for use in feed production in order to increase phosphate availability and reduce the need for synthetic phosphate fortification and the accompanying eutrophication issues. We have also demonstrated in this work that the strain TY1322 is able to release its phytase to the surrounding medium (depending on medium) in young viable cultures which, as discussed in our previous work (Hellstrom *et al*., 2015b,b), is not to be compared to the phytase release that occur in old cultures as an effect of dying and lysing cells. This early and high phytase release indicates a great potential application for reduced downstream processing in industrial crude phytase production, using this non‐GMO strain.

The purified phytase of TY13wt and TY1322 showed no repression in activity when assayed at high phosphate levels, meaning that the improved phytase activity at high phosphate levels seen by strain TY1322 is indeed an improved trait of the yeast and not a difference in the expressed phytase by this strain compared with TY13wt. Furthermore, the phytase produced by TY1322 maintains its activity (above 99%) after 10 s of incubation at 85°C. In the feed industry, heating to around 80–95°C is applied during the pelleting and held for a short time, from less than a minute to more than 2 min depending on factory and setup (Rasmussen, 2010). Although the phytase in our work was not stable at elevated temperatures for longer incubation times (60 s), it should be noted that during pelleting, various matrices and methods can be used in which the enzyme can be more or less protected from the heating (Rasmussen, 2010). To determine the thermotolerance and performance of the phytase from TY1322 for feed production applicability, tests need to be made in the real matrix under the real conditions.

The activity of the phytase from TY13wt and TY1322 in the presence of iron and copper was inhibited, while in the presence of magnesium and calcium, the activity appeared uninfluenced. The responses to the presence of metal ions are varying for various phytases, as for example Zhang and colleagues (Zhang *et al*., 2013) found activation by calcium, no effect of iron (Fe^3+^) and inhibition from magnesium and copper on fungal phytase, while Igamnazarov and colleagues (Igamnazarov *et al*., 1999) found activation by both magnesium and calcium, and inhibition by iron and copper on a bacterial phytase.

The proteomic analysis of the pooled purified samples of TY13wt and TY1322 shows a strikingly similar picture, with the three phytase sequences identified as the most abundant proteins in the preparations. Sequence coverages were also very similar between the TY13wt and TY1322 preparations (Table S1). However, in the absence of the gene sequencing data for each of the strains, proteomic experiment could not account for potential for mutations within the phytase sequences. As the characterization of the samples (performance at various pH, temperatures, in the presence of metal ions, etc.) showed the same results for both the TY13wt and TY1322 samples, the proteomic results further adds to our belief that the mutagenesis has resulted in an improvement of the yeast itself, rather than in the phytases produced by the yeast.

The wild‐type strain TY13wt and the improved strain TY1322 were further subjected for some phenotypic characterization. There was no major difference in growth performance during any of the investigated conditions for TY1322 compared with TY13wt. The strains were thermotolerant and grew up to 46°C; however, the growth was inversely correlated with higher temperatures and the highest growth occurred at 27–30°C. The strains were not sensitive towards ox bile up to 2% and grew well at low pH, showing growth even at pH 2. Furthermore, the strains showed high osmotic tolerance by growing in a medium of 60% glucose, and were also able to grow in ethanol concentrations up to 6% and lactic acid concentrations of 1%. The phenotypic traits of the yeast TY1322 indicates its ability to grow in a wide range of media and food matrices, and its robustness makes it interesting for application in industrial food and feed processes.

Cereal‐based fermented foods such as Togwa, from which the wild‐type strain in this work was initially isolated, are consumed containing viable cells. The potential for TY13wt and TY1322 to survive through the gastrointestinal tract (growth at low pH, in the presence of ox bile and at 37°C) indicates that they may function as probiotics, and may be able to mediate continued degradation of phytic acid from our meal also in the gastrointestinal tract after consumption. However, the probiotic potential of the strain TY1322 needs further investigation.

The phytate‐degrading capacity of strain TY1322 was 1.26 mmol IP_6_ per gram yeast and hour (Fig. 4). In a standard bread dough previously used (Andlid *et al*., 2004), 5.7 g yeast was added in a whole wheat flour dough with a total weight of 533 g, which means 0.011 g yeast per g dough. The dough was found to contain 9 μmol IP_6_/g. With the capacity of TY1322 found in the present study, degradation during 1 h of leavening would be 13.86 μmol IP_6_/g dough (1.26 mmol × 0.011 g), which is 54% more than the total amount in the dough. Expressed differently, with the rate found, TY1322 would degrade all IP_6_ in 39 min. However, several factors influence the yeast and enzyme activity in a dough, hence further studies are needed.

To conclude, yeast strain TY1322 and its phytase are shown to be promising candidates for application in both food and feed industries for production of goods with increased bioavailability of minerals and phosphate.

## Experimental procedures

### Strains and media

In this work, the strain *P. kudriavzevii* TY13wt, previously isolated from Tanzanian Togwa (Hellström *et al*., 2010), has been used as the parental strain for UV mutagenesis in order to isolate positive mutant strains.

For short‐term storage, yeasts were kept on YPD agar plates (10 g l^−1^ yeast extract, 20 g l^−1^ peptone, 20 g l^−1^ glucose and 15 g l^−1^ agar) at 4°C for up to 2 weeks. For long‐term storage, yeasts were kept in 15% glycerol solution at −80°C.

Determinations of survival rate were done by plating on YPD agar. Selection after mutagenesis was done on plates containing 6.9 g l^−1^ yeast nitrogen base with phosphate (Pi), YNB_wPi_ (Formedium), 20 g l^−1^ glucose and 0.05 g l^−1^ BCIP. Colonies possessing phosphatase activity turn blue on this medium after incubation due to BCIP, and colonies with higher activity could be visibly selected for further screenings.

For all liquid screenings, yeast nitrogen base without phosphate, YNB_w/oPi_ (Formedium), was used together with glucose and addition of various phosphate sources and other additives. For liquid screenings of phytate (IP_6_)‐degrading capacity, two phytate‐containing media (PM) without or with phosphate were prepared, referred to as **PM**_**NoPi**_ (YNB_w/oPi_ supplemented with 20 g l^−1^ glucose, 1 g l^−1^ IP_6_) and **PM**_**HPi**_ (YNB_w/oPi_ supplemented with 20 g l^−1^ glucose, 1 g l^−1^ IP_6_ and 3.5 g l^−1^ KH_2_PO_4_). For assessing secretion of non‐cell‐bound phytase in liquid media, a **SIM** (YNB_w/oPi_ supplemented with 20 g l^−1^ glucose and 10 g l^−1^ yeast extract) and a **NSIM** (YNB_wPi_ supplemented with 20 g l^−1^ glucose) were prepared.

In evaluations of biomass‐bound phytase activity, a Pi‐ and IP_6_‐rich medium was used (succinate buffer at pH 5.5 containing 20 g l^−1^ glucose, 3 g l^−1^ IP_6_, 3.5 g l^−1^ KH_2_PO_4_, YNB_w/o Pi_).

All incubations were carried out aerobically at 30°C.

### Determination of survival rate

*Pichia kudriavzevii* TY13 from −80°C storage were inoculated on YPD agar overnight before transfer to liquid YPD cultures. The pre‐culture was made in two steps, and cells were washed and resuspended in sterile 0.9% NaCl solution (saline) before inoculation to the experimental culture of 200 ml YPD to a starting optical density (OD, 600 nm) of 0.1. The OD was monitored during growth, and when the cultivation reached middle exponential phase, cells were harvested by centrifugation at 4000 *g* for 5 min. Cells were washed twice in sterile saline and resuspended in sterile saline to an OD of 1 (approximately 10^7^ cells ml^−1^). A volume of 50 ml was transferred into a sterile 500 ml beaker containing a magnetic stirrer and placed on a magnetic stirring table. The surface of the cell suspension was located 50 cm below the UV lamp (XX‐15M UV Bench Lamp, P/N 95‐0042‐15, 15 W and 230 V, from UVP, Upland, CA, USA), which was equipped with two UV‐C lamps of 254 nm (G15T8). Aliquots of 5 ml were withdrawn at times 0, 10, 20 and 30 s and immediately placed in the dark for 30 min. All work with cells after UV irradiation was done away from light. The withdrawn samples were then diluted to approximately 4000 cells ml^−1^, from which 100 μl were spread onto YPD plates. The plates were incubated in the dark for 48 h and the number of colonies was counted for determination of survival rate, using a non‐treated sample as control.

### Mutagenesis and selection of improved strains of *P. kudriavzevii* TY13

The mutagenesis was done according to the same procedure as for the determination of survival rate, with a total irradiation time of 18 s. The whole cell suspension was thereafter immediately placed in the dark for 30 min before diluting an aliquot of the cell suspension to approximately 3500 cells ml^−1^, from which 50 μl was streaked onto several agar plates containing BCIP. All plates were incubated in the dark for 48 h before colony investigation. Colonies showing stronger blue colour than the wild type, or that had an indication of blue halo formation were selected to be re‐streaked and further evaluated.

The mutagenesis was done in two consecutive rounds, the first one using TY13wt as parental strain, and the second one using the most prominent strain from round one, called TY1310, as parental strain.

### Screening methods for strains with improved phytate‐degrading capacity

Selected putative mutants, plus the parental strains *P. kudriavzevii* TY13wt and TY1310 from round 1 and 2 respectively, were pre‐cultured on YPD plates and then incubated overnight in 5 ml of the selected screening media before the screenings were conducted as described below. Due to the large number of putative mutants from the mutagenesis, the screenings were performed without replicates.

To assess the phytate‐degrading capacity in a phosphate‐free and a high‐phosphate media, respectively, all putative mutants were inoculated into a volume of 15 ml of PM_NoPi_ and PM_HPi_ respectively, to a starting OD of 0.3, and samples were withdrawn after 0, 3, 6 and 11 h of incubation. Samples were immediately made cell‐free by centrifugation at 4000 *g* and the cell‐free supernatants were mixed with HCl to a final concentration of 0.5 M to quench enzymatic reactions. Samples were then analysed for IP_6_ content by high‐performance liquid ion chromatography (HPIC) as described in our previous work (Qvirist *et al*., 2015) to determine the amount of degraded IP_6_. In brief, the supernatant and assay solution [1 g l^−1^ IP_6_ in acetate buffer (NaAc/HAc) pH 5] were mixed at 1:5 (vol:vol). Incubation was done at 37°C for 1 h with sampling at 0, 5, 10, 20, 30 and 60 min, with addition of HCl to a final concentration of 0.5 M to stop enzymatic reactions. All samples were kept at −20°C until the phytate analysis by HPIC.

To investigate the release of non‐cell‐bound phytases from the putative mutants, they were inoculated into a volume of 5 ml of SIM and NSIM respectively, to a starting OD of 0.5 and incubation was carried out for 9 h. The supernatants were then made cell‐free by centrifugation, and the cell‐free supernatants were used for the phytate degradation assay as described previously.

### Characterization of phytase‐active mutant strains TY1310 and TY1322

The superior strain from the first round of mutagenesis, annotated TY1310, was further used as parental strain in the second round of mutagenesis, generating the final strain annotated TY1322. After the screenings, TY1310 and TY1322, plus the original wild‐type strain (TY13wt) were used for further investigations as described below. In addition, the stability of the improved mutant strains TY1310 and TY1322 in terms of phytase activity was assessed by cultivating the strains for several generations on YPD medium and assessing the phytase activity.

The growth and IP_6_ degradation was assessed for the three strains TY13wt, TY1310 and TY1322 in the high‐phosphate medium, PM_HPi_, using triplicate cultures with incubation for 24 h. The IP_6_ degradation was additionally assessed also in phosphate‐free medium PM_NoPi_ but for 6 h of incubation. Samples were withdrawn for assessing growth (OD, 600 nm) and for determination of phytate concentration throughout incubation.

The sample to be used for phytate concentration determination was rapidly made cell‐free by centrifugation, and the enzymatic reaction was immediately stopped by adding HCl to a final concentration of 0.5 M, before analysis on HPIC.

To investigate the optimal pH and temperature for the biomass‐associated phytase, duplicate samples of washed biomass from overnight incubation in SIM were subjected to phytase assays at different pH and temperatures.

To study the phytase activity at different pH, TY13wt and TY1322 were inoculated into 5 ml SIM, from pre‐cultures of the same medium, and incubated overnight at 30°C. Biomass from 0.5 ml of each culture was harvested by centrifugation, washed twice in sterile milliQ, pelleted by centrifugation and then resuspended in 0.9 ml of assay buffer of different pH, containing 1 g l^−1^ IP_6_. The investigated pHs ranged from 2 to 9 (pH 2–3 glycine/HCl, pH 4–5 acetic acid/sodium acetate, pH 6 citric acid/NaOH, pH 7–8 Tris/HCl, pH 9 glycine/NaOH). Incubations were done at 40°C for 5 min, before the samples were made cell‐free by centrifugation and the enzymatic reactions were stopped by addition of HCl to a final concentration of 0.5 M and analysed using HPIC.

To study the phytase activity at different temperatures, cultures were prepared as for the pH tests, but biomass from 0.2 ml of each culture was used. Biomass was collected by centrifugation, washed twice in sterile milliQ and resuspended in 0.9 ml assay buffer of pH 3.5 containing 1 g l^−1^ IP_6_. Incubations were done for 3.5 min at the various temperatures, ranging from 30°C to 80°C. The samples were made cell‐free by centrifugation and the enzymatic reactions were stopped by addition of HCl to a final concentration of 0.5 M and analysed using HPIC.

To assess the biomass‐associated phytase activity of TY13wt and TY1322, a high P_i_ and high IP_6_ (3.5 g l^−1^ and 3 g l^−1^ respectively) medium was used. Two‐step pre‐cultures were made and the latter one was used to inoculate triplicate experimental cultures to a starting OD of 1. Two samples of 1 ml each were withdrawn from each culture at 6, 8, 10 and 15 h of incubation, to determine dry weight of the biomass and to assess the phytase activity from the biomass. Samples were harvested by centrifugation and cells were washed twice in sterile saline. For dry weight determination, cells were frozen and lyophilized. For phytase activity determination, cells were suspended in 1.3 ml of assay buffer with 1 g l^−1^ IP_6_ and incubated at pH 3.5 at 55°C, with sampling during 60 min of incubation. Samples were immediately made cell‐free by centrifugation and the enzymatic activity was stopped by addition of HCl to a final concentration of 0.5 M. Samples were analysed for phytate content on HPIC and the activity was expressed as mmol IP_6_ degraded per gram dry weight biomass and hour of assay.

### Purification and characterization of released phytase of TY13wt and TY1322

The two strains TY13wt and TY1322 were inoculated from −80°C stocks on YPD agar plates overnight. Thereafter, cells were inoculated into duplicate 25 ml cultures of SIM and incubated for 8 h, yielding a final OD of about 7.5. The cells were then used as inoculum to duplicate 300 ml SIM cultures with an initial OD of 0.07 for both strains. After 68 h of incubation, the cultures were centrifuged (4000 *g*) and filtered in 0.22 μm filter top unit (TTP) to remove all cells. The biomass‐free samples are referred to as *culture filtrate* samples. The culture filtrate samples were concentrated using Amicon filtration equipment, the membrane (Millipore cellulose, Bedford, MA, USA) having a cut‐off of 10 kDa, using magnetic stirring and overpressure of nitrogen gas. The buffer was simultaneously exchanged to succinate buffer of pH 5.5. This sample is referred to as *Amicon concentrate*. The Amicon concentrate was further concentrated using a spin filter (Macrosep, Pall filtration) with a 10 kDa cut‐off at 5000 *g*, and this sample is referred to as *spin filter concentrate*. The samples from those three steps were kept at 4°C until determination of protein content and phytase activity.

The protein content was determined (mg ml^−1^) by measuring the absorbance at 260 and 280 nm. The *SIM* medium, in which the phytase is expressed, contains yeast extract; hence, there is a potential presence of amino acids in this medium. As amino acids may interfere with the protein determination, the protein content was calculated with the formula 1.55*Abs_280_ − 0.76*Abs_260_ to adjust for possible presence of free amino acids (Stoscheck, 1990; Simonian, 2001). To maintain consistency, the calculation was applied for all protein determinations in this study.

The enzyme solution from the spin filter concentrate was used for size exclusion separation and fractionation using gel‐filtration on a Sephadex G75 gel column (1.5 × 20 cm, 13 cm bed height) with NaAc/HAc (0.1 M pH 4) containing 0.15 M NaCl as running buffer. A peristaltic pump (W‐M Alitea, Stockholm, Sweden) was used at a flow of 1 ml min^−1^ and fractions of 2 ml were collected using an automated fraction collector (Waters, Milford, MA, USA). All fractions were assessed for protein content, and all fractions containing detectable levels of protein were further investigated for phytase activity.

The phytase activity assay was performed by mixing each fraction at 1:10 (vol:vol) with the assay buffer (1 g l^−1^ IP_6_ containing NaAc/HAc buffer at pH 5), followed by incubation at 40°C for 5 or 10 min; thereafter, the enzymatic reactions were stopped by addition of HCl to a final concentration of 0.5 M. The samples were then analysed for IP_6_ content by HPIC, and the activity was expressed as U (μ mole degraded IP_6_/minute from the enzyme solution). The fractions containing phytase activity were pooled and stored at −20°C until further use.

For estimation of the molecular weight of the phytase, the pooled fractions of TY13wt and TY1322 phytases were mixed with loading buffer containing 10% v/v mercaptoethanol and boiled at 95°C for 5 min before being loaded onto a TGX 12% polyacrylamide gel (Bio‐Rad, Solna, Sweden) in a Tris/Glycine/SDS running buffer (Bio‐Rad). Bio‐Rad's precision Plus Kaleidoscope Standard (#161‐0375) was used as size ladder. The gel was run at 220 V for 23 min, stained with Coomassie blue C25 and scanned in a GS‐800 Calibrated Densitometer (Bio‐Rad).

The methodology used for concentration and purification of the phytase containing supernatants enable a very high degree of purification, even though a complete purification cannot be guaranteed as other same sized proteins may also be present in the final sample. However, in this study, we refer to the final pooled phytase sample as ‘purified’.

The purified enzyme samples were used for determination of optimal pH and temperature, by the same procedure as described for the cell‐associated phytase above.

The phytase activities of the purified phytase samples of TY13wt and TY1322 in the presence of various metal ions and in the presence of high phosphate levels were assessed based on the work by Igamnazarov and colleagues (Igamnazarov *et al*., 1999). The different metals used during assay was magnesium (Mg^2+^), calcium (Ca^2+^), copper (Cu^2+^) and iron (Fe^2+^) at either 1, 2 and 5 mM concentration. Metal chlorides were used as sources for the metals. The phytase assays were performed as described previously but at 55°C incubation temperature (found to be the optimal working temperature of this enzyme) and with one single sampling at 10 min. All experiments were done in duplicates. The phytate concentration in the differently treated samples were analysed by HPIC and compared with a positive control assay sample without addition of metals.

The pooled phytase of strain TY13wt and TY1322 were further assessed in duplicates for the activity in the presence of high levels of phosphate. The high‐phosphate assay mixture was prepared using 1 g l^−1^ IP_6_ and 3.5 g l^−1^ phosphate (KH_2_PO_4_) in acetate buffer at pH 5. As positive control, the same assay mixture was prepared, but without addition of phosphate. Incubation was done at 55°C for 10 min, and thereafter the amount of degraded phytate was determined from HPIC analysis.

The pooled phytase sample of strain TY1322 was assessed for its thermal tolerance, by incubating triplicate samples of 100 μl for 10 or 60 s in a water bath at 55°C, 65°C, 75°C, 85°C and 95°C respectively. After incubation, samples were transferred immediately to a cold (4°C) water bath. All samples were then used for phytase activity assay as described previously but at 55°C incubation temperature, with sampling after 10 min. The phytate concentration in the differently treated samples were analysed by HPIC and compared with the level found in the samples incubated at 55°C.

### Proteomics analysis of the purified enzyme samples of TY13wt and TY1322

The pooled protein samples of TY13wt and TY1322 (30 μg each) were digested with trypsin using the filter‐aided sample preparation method (Wiśniewski *et al*., 2009). Briefly, protein samples were reduced with 100 mM dithiothreitol at 50°C for 40 min, transferred on 30 kDa MWCO Pall Nanosep centrifugal filters (Pall Life Sciences, Ann Arbor, USA), washed with 8 M urea solution and alkylated with 10 mM methyl methanethiosulfonate in 50 mM TEAB and 1% sodium deoxycholate. Digestion was performed in 50 mM TEAB, 1% sodium deoxycholate at 37°C in two stages; the samples were incubated with 500 ng of Pierce MS‐grade trypsin (Thermo Scientific, Rockford, USA) overnight, then 500 ng more of trypsin was added and the digestion was for 3 h. The digested peptides were desalted using Pierce C‐18 spin columns (Thermo Scientific, Rockford, USA), the solvent was evaporated and the peptide samples were reconstituted in 3% acetonitrile, 0.1% formic acid solution for LC‐MS/MS analysis.

For the LC‐MS/MS analysis, each sample was analysed on Q Exactive mass spectrometer (Thermo Fisher Scientific, Bremen, Germany) interfaced with Easy‐nLC II nanoflow liquid chromatography system. Peptides were trapped on the C18 trap column (200 μm × 3 cm, particle size 3 μm) separated on the home‐packed C18 analytical column (75 μm × 30 cm, particle size 3 μm) using the gradient from 7% to 27% B in 25 min, from 27% to 40% B in 5 min, from 40% to 80% B in 5 min at the flow rate of 200 nl min^−1^; solvent A was 0.2% formic acid and solvent B was 98% acetonitrile and 0.2% formic acid. Precursor ion mass spectra were recorded at 70 000 resolution. The 10 most intense precursor ions were fragmented using HCD at collision energy setting of 30 spectra and the MS/MS spectra were recorded at 35 000 resolution. Charge states 2–6 were selected for fragmentation, and dynamic exclusion was set to 30 s.

For identification of proteins, a database search was performed using Proteome Discoverer version 1.4 (Thermo Fisher Scientific, Waltham, USA). Sequence database for *P. kudriavzevii* (August 2016, 6873 sequences) was downloaded from Uniprot repository (Proteome ID UP000029867), and the phytase sequences were manually identified in the database and marked as the ‘putative phytases’. Mascot 2.3.2.0 (Matrix Science, London, United Kingdom) was used as a search engine with precursor mass tolerance of 15 ppm and fragment mass tolerance of 0.02 Da. One missed cleavage was allowed; mono‐oxidation on methionine was set as a variable modification, and methylthiolation on cysteine was set as a fixed modification. Target/decoy approach was used to refine the identification results, and target false discovery rate of 1% was used as a threshold to filter the confidently identified peptides.

### Phenotypic characterization of yeast strains TY13wt and TY1322

The wild‐type strain TY13wt and strain TY1322 were investigated for growth at different cultivation conditions. All cultivations were done in triplicates in 96‐well micro plates. The cultivation volumes were 195 μl and the inoculation volume from overnight cultures was 5 μl, yielding a starting OD about 0.2.

The strains were tested for growth in YPD at pH 2, 3 and 4.8, in YPD with ox bile at 0.5%, 1% and 2% (w/v), all at 37°C and 150 r.p.m. orbital shaking. The growth was also assessed in YPD (natural pH 6.5) at 27°C, 37°C, 42°C, 46°C, 48°C and 50°C. The growth was assessed by measuring the optical density at 630 nm after 3 days of incubation at 30°C (or at the test temperature) with shaking at 150 r.p.m.

Utilization of different sugars was tested in micro well plates in duplicates, using a base of yeast extract (10 g l^−1^) and peptone (20 g l^−1^) and addition of 20 g l^−1^ of either glucose, xylose, lactose, maltose, mannitol, sucrose or arabinose. Furthermore, growth in the presence of lactic acid or ethanol was tested duplicates in a medium containing yeast extract (10 g l^−1^) and peptone (20 g l^−1^) with addition of 1%, 6% or 12% (vol/vol) of lactic acid or ethanol. To test the osmotic tolerance, YPD was prepared using 50% or 60% of glucose. The growth was assessed by measuring the optical density at 630 nm after 2 days of incubation at 30°C with shaking at 150 r.p.m.

The resistance towards oxidative stress was investigated by spreading a dense liquid yeast culture of each strain on small YPD plates, allowing the liquid cell suspension to absorb, and thereafter placing a filter paper (d = 5 mm) soaked in hydrogen peroxide (H_2_O_2_) in the centre of the plate. By measuring the length from the centre of the filter papers to the yeast growth zone border after 48 h of incubation at 27°C, the relative resistance to oxidative stress could be compared between the strains.

## Supporting information


**Fig. S1.** Protein content (lines marked with circles) and phytase activity (lines marked with cross).

**Fig. S2.** Phytase activity for purified phytase solution at different temperatures.

**Table S1.** Top 3 proteins identified in the TY13wt and TY1322 purified samples using LC‐MS analysis.
