# Supplementary figures and images for: Strain improvement of Pichia kudriavzevii TY13 for raised phytase production and reduced phosphate repression
Source: Microb Biotechnol. 2016 Oct 28;10(2):341–53. doi: 10.1111/1751-7915.12427 (PMC5328827; doi:10.1111/1751-7915.12427)

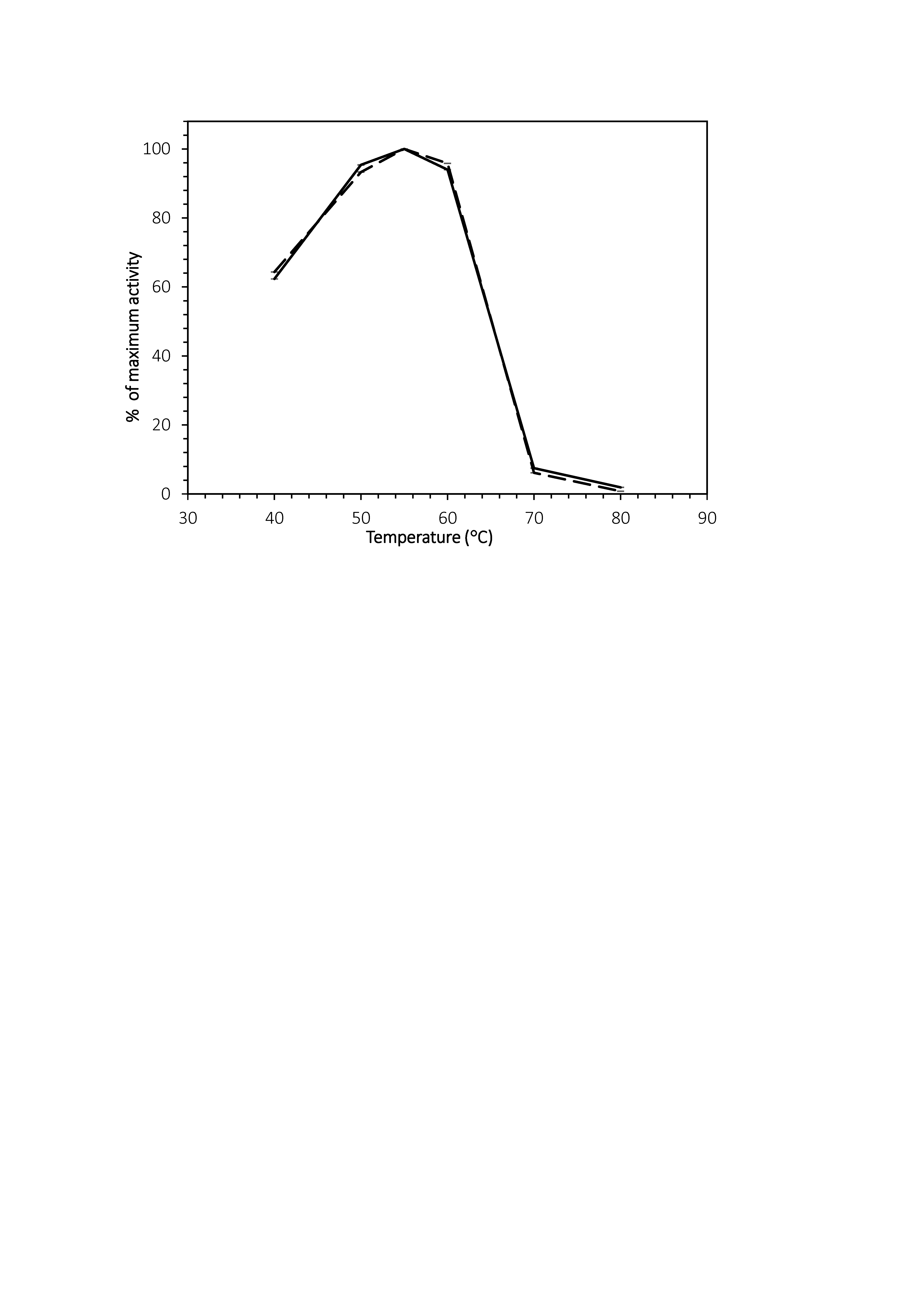

Supplement: Supplementary file 2 — Fig. S2. Phytase activity for purified phytase solution at different temperatures. [file MBT2-10-341-s002.tif]

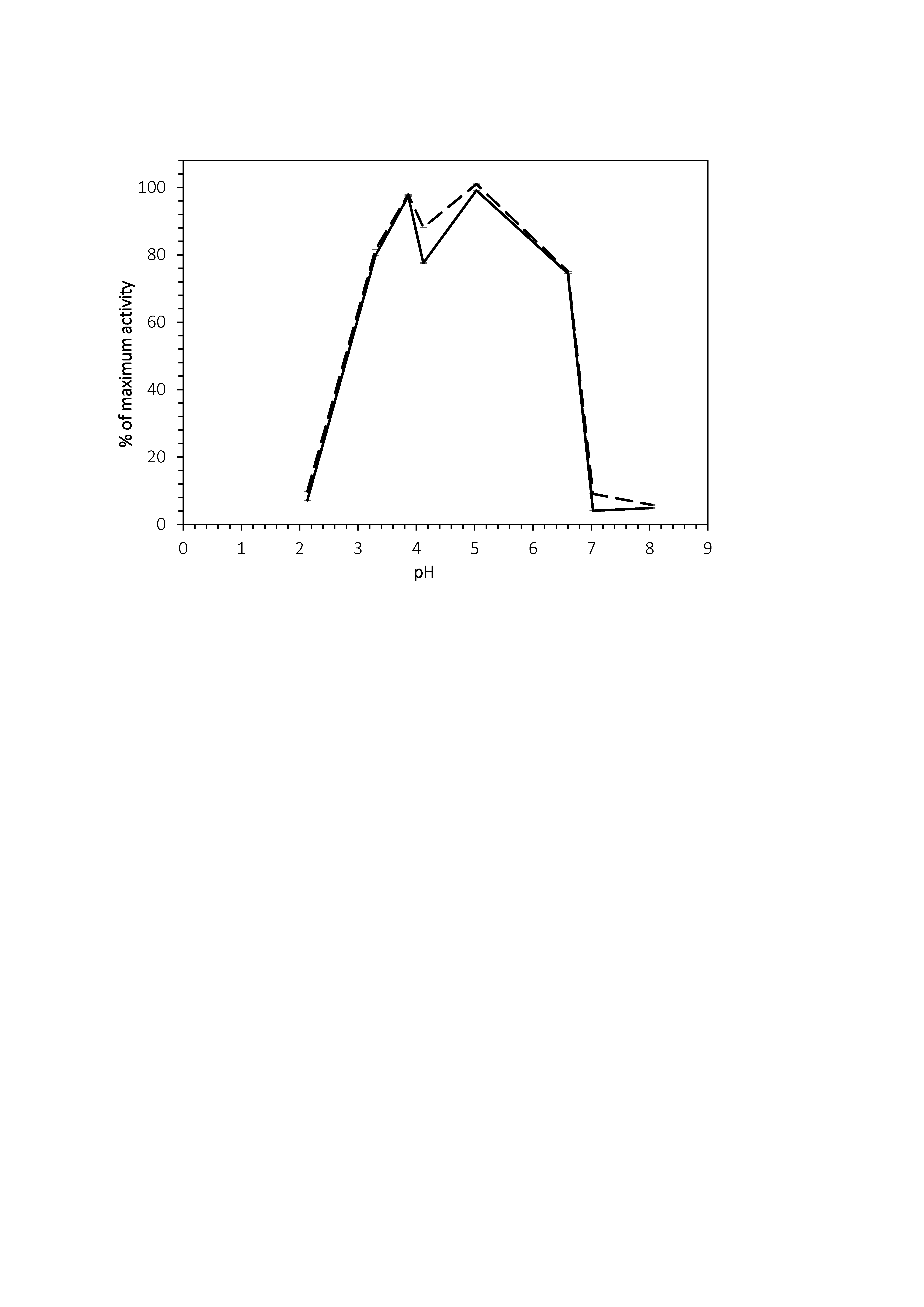

Supplement: Supplementary file 3 [file MBT2-10-341-s003.tif]
