# Supplementary material for: Strain improvement of Pichia kudriavzevii TY13 for raised phytase production and reduced phosphate repression
Source: Microb Biotechnol. 2016 Oct 28;10(2):341–53. doi: 10.1111/1751-7915.12427 (PMC5328827; doi:10.1111/1751-7915.12427)
Supplement: Supplementary file 4 — Table S1. Top 3 proteins identified in the TY13wt and TY1322 purified samples using LC‐MS analysis. [file MBT2-10-341-s004.docx]

**Table** **S1**. Top 3 proteins identified in the TY13wt and TY1322 purified samples using LC-MS analysis. Proteins were digested with trypsin, identification was performed using Mascot search engine in the *P. kudriavzevii* Uniprot database. Only the peptides that pass 1% FDR threshold were used for identification.

| **Sample TY13wt** | | | | | |
| --- | --- | --- | --- | --- | --- |
| **Uniprot Accession** | **Protein Description** | **Mascot Identification Score** | **Sequence Coverage (%)** | **Peptides** | **Unique peptides** |
| A0A099NYB3 | Putative phytase GN=JL09_g3064 | 6036.76 | 37.08 | 22 | 13 |
| A0A099P0J1 | Putative phytase GN=JL09_g3065 | 4919.18 | 27.86 | 16 | 8 |
| A0A099NYX9 | Putative phytase GN=JL09_g3063 | 3180.21 | 19.37 | 10 | 1 |
| **Sample TY1322** | | | | | |
| **Uniprot Accession** | **Protein Description** | **Mascot Identification Score** | **Sequence Coverage (%)** | **Peptides** | **Unique peptides** |
| A0A099NYB3 | Putative phytase GN=JL09_g3064 | 5966.89 | 36.53 | 22 | 14 |
| A0A099P0J1 | Putative phytase GN=JL09_g3065 | 5285.00 | 27.31 | 15 | 8 |
| A0A099NYX9 | Putative phytase GN=JL09_g3063 | 3121.17 | 23.25 | 11 | 3 |
