# Supplementary material for: Strain improvement of Pichia kudriavzevii TY13 for raised phytase production and reduced phosphate repression
Source: Microb Biotechnol. 2016 Oct 28;10(2):341–53. doi: 10.1111/1751-7915.12427 (PMC5328827; doi:10.1111/1751-7915.12427)
Supplement: Supplementary file 5 [file MBT2-10-341-s005.docx]

**Supplementary Figure S1**. Protein content (lines marked with circles) and phytase activity (lines marked with cross) on the supernatant of TY13wt (solid lines) and TY1322 (dashed lines), during fractionation and separation on Sephadex G75 gel column.

**Supplementary Figure S2**. Phytase activity for purified phytase solution at different temperatures from 40°C to 80°C (on the left), and at different pH from 2 to 8 (on the right), strain TY13wt is presented by a solid line and TY1322 by a dashed line.
